# Camouflage

**Published:** 1918-03-02

**Authors:** 


### NURSING AFFAIRS IN IRELAND.

### Camouflage: Ierne's Interesting Challenge.

AMONGST the unconsidered trifles which the war has brought us is an addition to our vocabulary. One particularly interesting word which is likely to remain is "camouflage." The other day most of us heard it for the first time: now we wonder how we got along without it in the brave days of old. When a doctor gilds a pill: when a detective wears false whiskers: when the cook dishes up last week's bone so that the ghost of its defunct owner would not recognise it, this one word describes all the processes. Camouflage is a bluff—a disguise designed to deceive. Probably the biggest camouflage of the war was our fleet of wooden, gunless battleships that hung round the curtains of the North Sea, which tantalised and fooled the enemy for many months. If I mistake not, we borrowed the idea from the famous captain of the *Emden*, who at an hour's notice was able to rig up his ship to look like any craft on the sea except itself. Camouflage, reader, you will accordingly observe, has become a recognised artifice of warfare, and is occasionally very useful for its purpose.

I hope that my Editor will not regard me as having gone off my balance for sending him this terminological disquisition in these days of paper famine. My apology is that I have been looking up some back numbers containing, amongst other things, Miss Eden's correspondence relating to the College of Nursing, and every time I arrive at her signature I find myself expecting to see written thereunder, not "Hon. Sec. to the National Union of Trained Nurses," but "Camouflage artist." There is no doubt that, in her own field of operations, Miss Eden rivals the captain of the *Emden* for skill and for courage.

### THE DEMOCRATIC STUNT.

Miss Eden's favourite paint-pot is labelled "Democracy," and the artist can use her brush so as to make a Prussian swashbuckler look for all the world a Bolshevik, and *vice versa*, while an army of nurses look on in bewilderment, if not in dismay. I have, I confess, been more than once bewildered by Miss Eden's artifices, and, again, I have even wondered if she herself is the victim of her art. Does Miss Eden really believe what she says about the College of Nursing, and does she likewise believe what she says about the institutions whose cause she champions? I should like, if possible, to put this to a simple and practical test.

In a letter to the *Manchester Guardian*, written this month (February), Miss Eden refers to the Scottish Nurses' Association (for Scotland), the Irish Nurses' Association (for Ireland), and the National Union of Trained Nurses (for England) as "democratic societies which have done a great deal for nurses educationally, and to advance their cause in every way." Does Miss Eden believe this to be true of the Irish Nurses' Association? I should very much like her to add one more letter to the many which have come from her pen, and, without any beating round the bush, to prove her statement. It may perhaps be difficult to demonstrate absolutely whether or not the Irish Nurses' Association has "done a great deal for nurses educationally," or has "advanced their cause in every way," whatever meaning their "cause" may, in Miss Eden's interpretation, be intended to convey. In any case it does not matter very much what they have done or left undone, so far as this argument is concerned. There can be no question, however, whether this Society is or is not in its constitution democratic. Of course Miss Eden may have one meaning for "democratic," and mine may be quite different. The Kaiser professes to believe that under his benign All-Highest rule the Poles are now enjoying the benefits of self-determination, but it is open to doubt that the Poles hold the same opinion. The test which I propose to apply, and which I challenge Miss Eden to accept or reject, is to undertake to establish her claim, subject to a penalty of £5 to be paid to the British Red Cross. If Miss Eden succeeds, I on my part agree to pay a like penalty to the same cause. Is the offer a fair one? If Miss Eden agrees, we can easily fix on an impartial umpire—some acknowledged leader of democratic opinion who will have little difficulty in getting under the paint. I am not intimately acquainted with the constitution and inner working of the two other societies to which Miss Eden refers, but her standard of democracy, judging from her citing of the I.N.A. as an ideal, is not reassuring.

### THE PAWNS IN THE GAME.

I am not in the smallest degree ambitious to achieve a controversial victory over Miss Eden. I am simply conscious of the fact that a great army of the most deserving women in His Majesty's realms are being made pawns in a game which is not to their advantage—women who can never hope to advance their interests save by unified action. The Irish Nurses' Association is, in my opinion, an impudent autocracy. Whether this opinion is justified or not can be determined by reference to its constitution. The method of appointing its executive and the voting powers of its members are in black-and-white, and I have a copy in my possession. Miss Eden champions its claims in the name of Democracy. A call is made to the nursing profession to postpone their Registration Bill until this Association is recognised by the State. The Irish nurse is at the same time the worst paid in the kingdom, and continues to offer up her life in the cause of involuntary charity despite the fact that the Association has been in existence for eighteen years, always endeavouring, as alleged, to educate her and to advance her cause.

The time has come when all concerned should endeavour to get down to bedrock facts, and it is with the object of helping thitherward that I throw down my challenge.

IERNE.

